# AIB1/SRC-3/NCOA3 function in estrogen receptor alpha positive breast cancer

**DOI:** 10.3389/fendo.2023.1250218

**Published:** 2023-08-30

**Authors:** Amber J. Kiliti, Ghada M. Sharif, Mary Beth Martin, Anton Wellstein, Anna T. Riegel

**Affiliations:** ^1^ Department of Oncology, Lombardi Comprehensive Cancer Center, Georgetown University, Washington, DC, United States; ^2^ Department of Biochemistry, Molecular and Cellular Biology, Georgetown University, Washington, DC, United States

**Keywords:** AIB1, SRC3, Ncoa3, estrogen receptor, breast cancer, endocrine therapy, transcriptional regulation

## Abstract

The estrogen receptor alpha (ERα) is a steroid receptor that is pivotal in the initiation and progression of most breast cancers. ERα regulates gene transcription through recruitment of essential coregulators, including the steroid receptor coactivator AIB1 (Amplified in Breast Cancer 1). AIB1 itself is an oncogene that is overexpressed in a subset of breast cancers and is known to play a role in tumor progression and resistance to endocrine therapy through multiple mechanisms. Here we review the normal and pathological functions of AIB1 in regard to its ERα-dependent and ERα-independent actions, as well as its genomic conservation and protein evolution. We also outline the efforts to target AIB1 in the treatment of breast cancer.

## Introduction

Breast cancer (BC) is the most common newly diagnosed cancer in women as of 2022 and one of the leading causes of cancer-related deaths (reviewed in 1). Breast cancer can be classified into main subtypes based on hormone receptor and human epidermal growth factor 2 (HER2) status (reviewed in 2). Hormone receptor positive BC expresses the estrogen receptor (ER) and/or the progesterone receptor (PR) which are targets for endocrine therapy. The majority of diagnosed BC cases are ER positive (3). These ER positive cancers are treated with endocrine therapies that quench estrogen production or target the ER directly but resistance is a common occurrence which leads to relapse and disease progression (reviewed in 4).

Steroid nuclear receptors, including the ER, are transcription factors (TFs) that regulate the expression of target genes when bound by their ligand. Steroid receptor coactivators are proteins that bind to steroid nuclear receptors and potentiate their transcriptional activity. AIB1/SRC-3/NCOA3 (Steroid Receptor Coactivator 3/Nuclear Receptor Coactivator 3) is a potent coactivator of the ER and plays a major role in normal physiology and human disease (reviewed in 5). It has been shown to be a key molecule involved in breast cancer malignant progression and in resistance to endocrine therapy. Therefore, understanding AIB1 function in BC is of value and targeting AIB1 in human BC is of clinical interest.

Here we summarize current knowledge on the structure, evolution and biological effects of AIB1 in the normal mammary gland and in ER positive and negative BC development and progression, providing insights into the rationale for targeting AIB1 in endocrine therapy resistant cancer.

## Estrogen receptor

**Figure 1:**
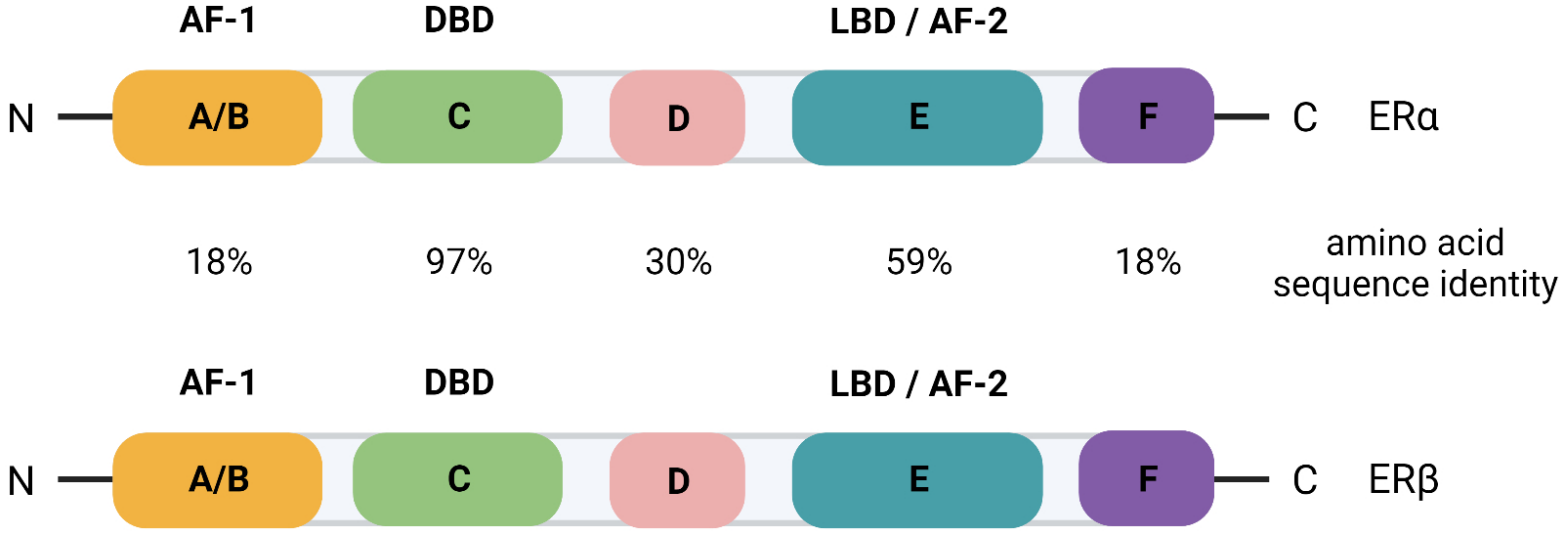
Schematic of the domain organization of ERα and ERβ with the percent amino acid sequence identity indicated for each domain. AF, transactivation function; DBD, DNA binding domain; LBD, ligand binding domain.

The ER is a nuclear receptor that is activated by its steroid ligand 17-β estradiol (E2). Many of the actions of estrogens in the breast are mediated by two isoforms of the estrogen receptor, ERα and ERβ. The mitogenic actions of the hormone are mediated by ERα while the antimitogenic actions are mediated by ERβ (reviewed in 6). ERα belongs to the superfamily of nuclear receptors (7) and, similar to other receptors, ERα is divided into regions A through F (**Figure 1**) (8). The N-terminal A/B region contains the transactivation function-1 (AF-1) domain. It is involved in protein-protein interactions and plays an important role in ligand dependent and independent activation of the receptor. Region C is the DNA binding domain (DBD) and consists of two zinc finger motifs connected by a short flexible amino acid linker. The hinge region, region D, plays a role in dimerization of the receptor. Region E is the hormone, or ligand, binding domain (LBD) and contains the ligand binding pocket and the transactivation function-2 (AF-2) domain. Region E is responsible for ligand dependent activation of the receptor and binds coactivators and corepressors. In the absence of hormone, ERa is associated with heat shock proteins, including hsp90 and hsp70, and immunophilins that maintain the receptor in high affinity ligand binding and render the receptor inactive by inhibiting DNA binding, dimerization, and cofactor binding (9, 10). Upon hormone binding, ERα is phosphorylated on serines in the A/B region that increases the activity of the AF-1 domain (11, 12) and a conformational change occurs in the LBD that results in the dissociation of heat shock proteins and the formation of the AF-2 domain (13). The ER then binds to estrogen-responsive elements (EREs) within the genome to regulate the expression of target genes. The ER can also indirectly interact with DNA through tethering to other TFs.

The ERα (*ESR1* gene) isoform stimulates proliferation and survival of breast tissue and has unequivocally been established as a driver of breast cancer carcinogenesis. More than 75% of all newly diagnosed BCs are classified as ER+ (3). Inhibiting ER signaling with anti-estrogen therapy has greatly improved the survival of patients with ER+ BC, however, some tumors display intrinsic resistance with no initial response and still many others develop acquired resistance (reviewed in 4). Hence, overcoming resistance remains a major challenge in treating ER+ BC.

The function of ERβ (*ESR2* gene) in breast cancer progression is largely unknown. However, a recent study has probed over 3000 primary breast tumors for RNA levels of *ESR2* and found that, although its overall expression is low, *ESR2* is associated with better overall survival (14). The domain organization of ERβ is similar to ERα and there is a high degree of amino acid sequence identity (**Figure 1**) (15). AIB1 can function as a coactivator of both ER isoforms (16), likely because there is conservation of the AF-2 domain where coactivators and corepressors bind.

## p160 family of coactivators

Competition experiments involving steroid nuclear receptors such as the ER revealed transcriptional interference, suggesting that transcriptional activation was facilitated by limiting factors (17). In 1995, Oñate et al. discovered the Steroid Receptor Coactivator 1 (SRC-1) from a yeast two-hybrid screen as a protein that interacted with the ligand-binding domain of the human PR (18). SRC-1 was shown to enhance receptor-mediated transactivation of the PR as well as augmenting receptor-mediated transcription of several other steroid receptors including the ER and the glucocorticoid receptor. Shortly thereafter, SRC-2/GRIP-1/TIF2 was discovered (19–21). The AIB1 (Amplified in Breast Cancer 1) (nuclear coactivator 3, *NCOA3*) gene was cloned in parallel by several laboratories and therefore has many names, including SRC-3, ACTR (activator of thyroid and retinoic acid receptor), TRAM-1 (thyroid receptor activator molecule 1), RAC-3 (RAR-associated coactivator 3), and p/CIP (p300/CBP cointegrator associated protein) (22–27).

The three homologous SRC family coactivators are ubiquitously expressed and share 50-55% sequence similarity. The resulting proteins are approximately 160 kDa (reviewed in 28). They have several conserved domains and therefore function similarly to activate transcription. The most conserved domain is the amino-terminus bHLH-PAS (basic helix loop helix- Per Arnt Sims) domain (29). The SRC coactivators also contain a central nuclear receptor domain (NRD) that is comprised of three alpha-helical LXXLL (where L is leucine and X is any amino acid) motifs which are essential for their interactions with nuclear receptors (30, 31). The sequences flanking these motifs are important for nuclear receptor selectivity. Finally, there are two activation domains (AD1 and AD2) that recruit co-coactivators at the carboxyl-terminus of the proteins (reviewed in 28). Many factors are known to interact with these domains (reviewed in 5).

Coregulators serve to bridge nuclear receptors to the transcription machinery and in general do not bind directly to DNA. They also modify chromatin and recruit secondary coactivators or corepressors to modulate transcription (reviewed in 32). SRC-1 and AIB1 have their own weak acetyltransferase activity in their C-terminal activation domains (24, 33). However, recruitment of CBP/p300 through the CID (CBP/p300 interaction domain) within the AD1 domain provides strong histone acetylase activity. Notably, although SRC proteins were initially discovered to activate transcription of nuclear receptors, they can also activate transcription of other TFs including NF-κB (nuclear factor kappa B), AP-1 (activator protein-1) and STAT6 (34–36).

## Transcriptional function of AIB1

**Figure 2:**
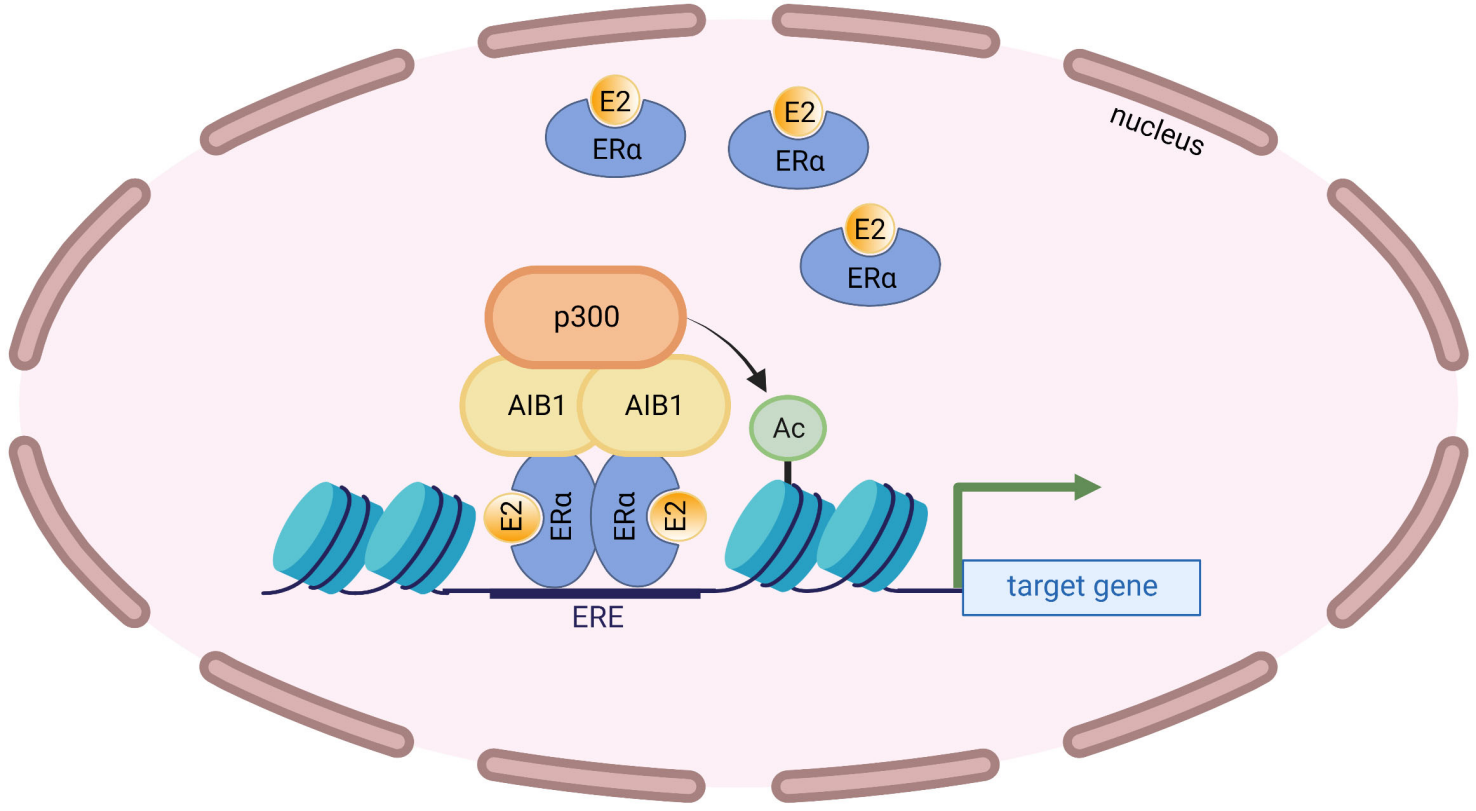
AIB1 interacts with the ligand-bound ERα at EREs and recruits the histone acetyltransferase p300. p300 acetylates nearby histones that opens up the chromatin leading to transcriptional activation.

AIB1 has been known to augment the transcriptional activity of the ERα in a ligand-dependent manner (22, 37). In fact, mass spectrometry revealed that AIB1 was the most enriched protein to immunoprecipitate with ERα after E2 treatment (38). Coactivator protein complexes are recruited to ligand-bound ERα once the nuclear receptor binds to EREs and SRC proteins have been shown to be among the earliest recruited factors (39). AIB1 facilitates transcription in part by recruiting chromatin-remodeling histone acetyltransferases such as p300 that relax chromatin from histones, leading to a permissive chromatin state (24) (**Figure 2**). The methyltransferase CARM1 (coactivator-associated arginine methyltransferase 1) can also be recruited following AIB1 and p300 which leads to reorganization of the complex, histone methylation and enhanced target gene transcription (40). Furthermore, AIB1 can regulate protein levels of ERα by recruiting the ubiquitin-proteosome machinery and, consequently, ERα levels are stabilized when AIB1 levels are decreased (41).

Enhancers are regulatory DNA sequences that can act in *cis* over long distances to stimulate gene transcription (42, 43). TFs bind to enhancer elements and, when in close proximity to their associated promoters, activate transcription (reviewed in 44, 45). Enhancers are brought close to their promoters through chromatin looping (46). The use of the novel looping assay developed by Panigrahi et al. to probe enhancer-promoter contacts *in vitro* revealed that AIB1 is a critical factor in looping that supports chromatin interactions at the ERα-regulated *GREB1* gene (47). The enhancer and promoter of *GREB1* in MCF-7 human breast cancer cells is held in a ready state through contacts with AIB1 while in an E2-depleted environment. AIB1 is bound to intronic sequences located downstream of the *GREB1* transcription start site. Upon E2 treatment, ligand-bound ERα is recruited to the gene loci and there is rapid reorganization that leads to productive transcription. Intriguingly, in this model, AIB1 is in direct contact with DNA and these binding sites were found to be necessary for gene transcription by ERα. This discovery indicates that AIB1 facilitates ERα-regulated gene transcription by modulating dynamic chromatin interactions. AIB1 is also known to interact directly with the pioneer factor FOXA1 or with TFs whose binding to DNA is facilitated by FOXA1 (48). Ligand activated ERα then competes with FOXA1 for the limited amounts of AIB1 found in the cell and this loss of AIB1 at FOXA1 loci leads to the early down-regulation of genes following E2 treatment. This data helps explain how AIB1 can have broad impacts on gene transcription and supports a model of physiological squelching.

**Figure 3:**
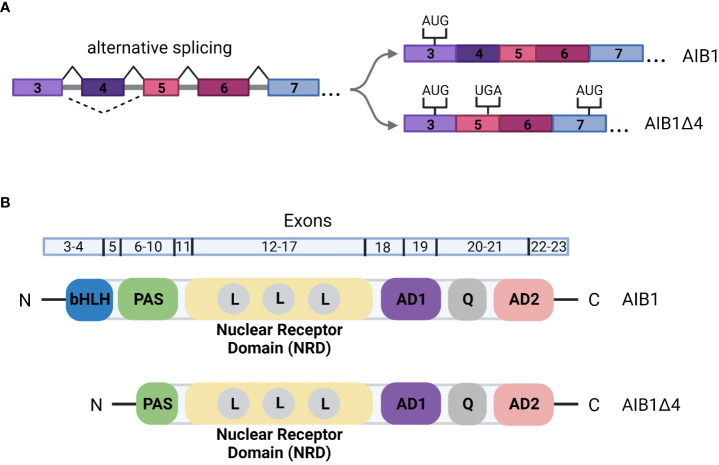
**(A)** Alternative splicing of *AIB1/NCOA3*. Skipping of exon 4 leads to a stop codon in exon 5. An alternative translation start site is then utilized in exon 7. The remainder of the gene is transcribed as in full-length AIB1. **(B)** Schematic of the domain organization of AIB1 and AIBΔ4. Exons that correspond to the domains are indicated above the structure. bHLH, basic helix-loop-helix; PAS, PER-ARNT-SIM; L, LXXLL motif; AD, activation domain; Q, glutamine rich region.

The gene for AIB1, *NCOA3*, is subject to alternative splicing. One such alternative splicing event produces an N-terminally truncated form of the full-length protein known as AIB1Δ4. AIB1Δ4 is generated by skipping of exon 4 (**Figure 3A**). This splice event leads to an in-frame stop codon in exon 5 and the usage of an alternative start codon in exon 7 (49, 50). The final protein product is 223 amino acids shorter from the N-terminus and is missing the bHLH-PAS domain (**Figure 3B**). Given the exclusion of this domain, AIB1Δ4 could interact with a distinct set of molecules or lose the ability to recruit other coregulators; for example, the putative tumor suppressor ANCO1/ANKRD11 that is recruited to gene loci by binding to the full length AIB1 is unable to bind to AIB1Δ4 (51). The transcription induced by the ER and PR is significantly increased with AIB1Δ4 compared to full-length AIB1 and AIB1Δ4 is associated with twice as much CBP/p300 (50). The AIB1Δ4 truncated protein is found at significantly higher levels in tumor samples compared to normal tissue and studies have shown that AIB1Δ4 increases the metastatic capabilities of BC cells (49, 52). For example, analysis of breast and pancreatic cancer cell lines showed a 2- to 4-fold higher abundance of AIB1Δ4 mRNA in metastatic lines compared to their less metastatic counterparts (50). In addition, samples from patients with high grade ductal carcinoma *in situ* (DCIS) had higher levels of AIB1Δ4 mRNA than lower grade samples (52). CRISPR engineering of human breast epithelial cells to only express the AIB1Δ4 isoform uncovered an “enabler” effect through cell-cell crosstalk with isogenic full-length AIB1 expressing cells. Interestingly, *in vivo* cancer cell invasion and metastasis was enhanced when cells expressing full-length AIB1 were mixed with a subpopulation of AIB1Δ4 expressing cells, demonstrating AIB1Δ4’s novel role in breast cancer progression (52, 53).

## Post-translational modifications and signaling pathways that regulate AIB1 function

AIB1 is phosphorylated at several serine and threonine residues that impact its activity and protein binding partners (reviewed in 54). Six phosphorylation sites were shown to be required for coactivation of the ER and AR (55). In addition, tyrosine phosphorylation by c-Abl (v-Abl Abelson murine leukemia viral oncogene homolog 1) tyrosine kinase at position Y1357 of AIB1 has been demonstrated to modify AIB1’s interaction with CARM1, p300 and ERα (56). Phosphorylation by Cdk1 impacts the subcellular distribution of AIB1 during mitosis (57) and phosphorylation at serine 857 by metabolic enzyme 6-phosphofructo-2-kinase/fructose-2,6-bisphosphatase 4 (PFKFB4) enhanced AIB1 transcriptional activity (58). Other prominent molecules including ERK, JNK, p38, IKK and PKA have all been shown to phosphorylate AIB1 leading to signal convergence and the ability of AIB1 to respond to the dynamic conditions of the cell (55, 59). Sumoylation of AIB1 was shown to diminish the transcriptional activity of AIB1 and E2 treatment led to a decrease in AIB1 sumoylation (60). Consistent with this finding, phosphatases PDXP, PP1 and PP2A are negative regulators of AIB1 transcriptional coregulatory activity (61). Furthermore, phosphorylation, methylation and ubiquitination events control AIB1 protein stability and turnover (62–64).

## Genomic and protein conservation of AIB1 across species

**Figure 4:**
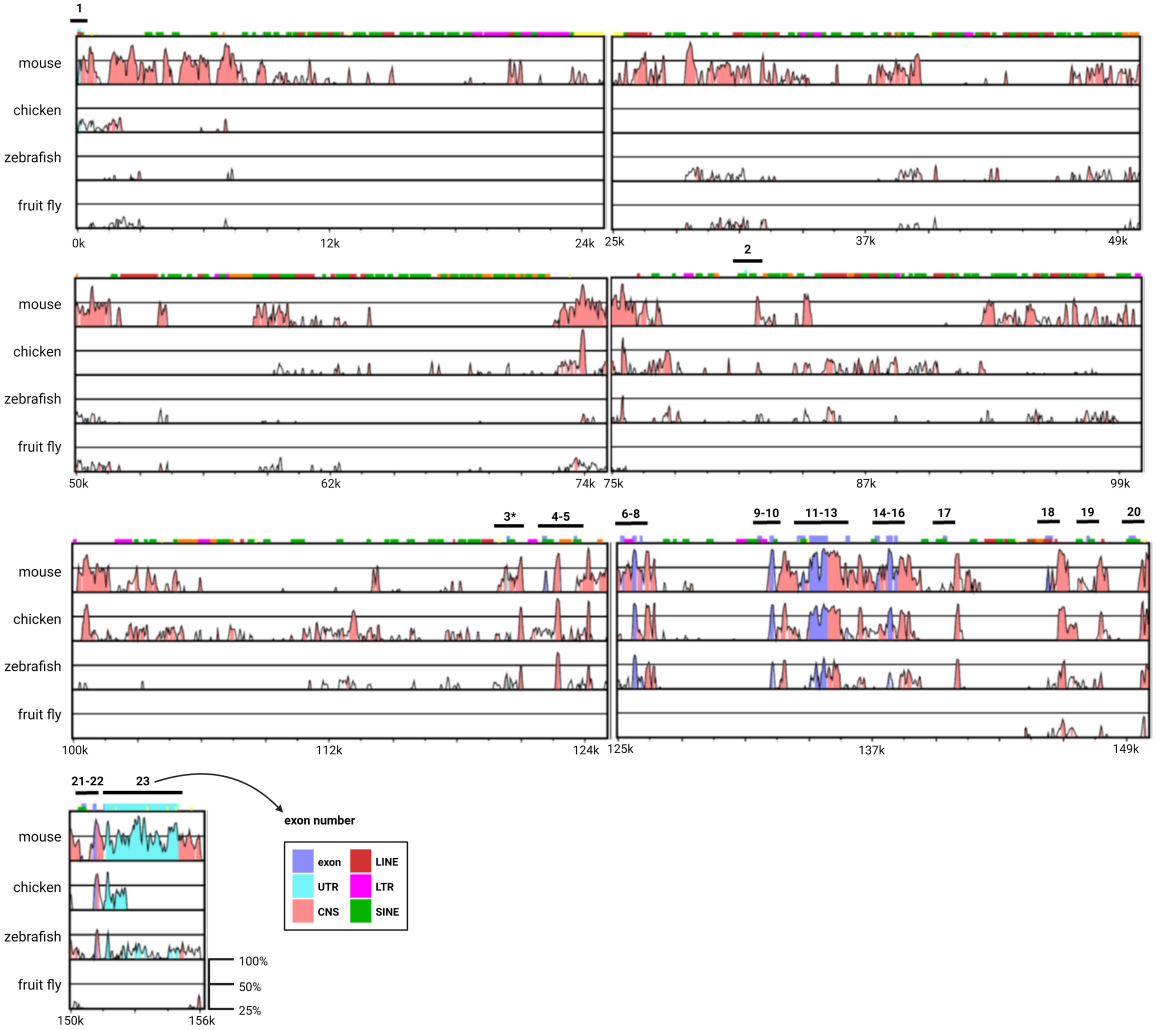
Genomic architecture of the *NCOA3* gene. mVISTA global alignment of mouse, chicken, zebrafish and fruit fly orthologs to human *NCOA3*. X-axis human sequence position. Y-axis percent conservation. Pink regions correspond to conserved non-coding sequences (CNS), purple regions indicate exons and light blue regions indicate untranslated regions (UTRs). LINE, long interspersed nuclear elements; LTR, long terminal repeat; SINE, short interspersed nuclear elements; *human exon containing translation start codon.

Vertebrate models have greatly enhanced our understanding of the physiological and pathological functions of AIB1 in mammary cancer. This extrapolation is possible in part because human and mouse AIB1 have high levels of genomic and protein conservation. Human *NCOA3* consists of 23 exons separated by introns of varying lengths. The largest of these introns is over 81 kb (intron 1-2), while the smallest is just 102 bp (intron 15-16). Exon lengths are more homogeneous. With the exception of exon 23, which contains almost exclusively 3’ UTR, human *NCOA3* exon lengths range from 79 bp (exon 2) to 872 bp (exon 12). To review the conservation of the genomic architecture of *NCOA3*, alignment of four divergent species to the genomic reference sequence of human *NCOA3* using the global-alignment program mVISTA (65, 66) was done (**Figure 4**). The peaks and valleys graph indicates the percent conservation for mouse, chicken, zebrafish and fruit fly respectively across the human *NCOA3* sequence. Human exonic sequences are well conserved in mouse, chicken and zebrafish, particularly after exon 3, which harbors the start codon for human, mouse, chicken and fruit fly. There are several regions in the mouse, and to a lesser extent in the other species, that have high levels of conservation throughout their intronic sequences. Additionally, the 3’ UTR is highly conserved between human and mouse (exon 23).

**Figure 5:**
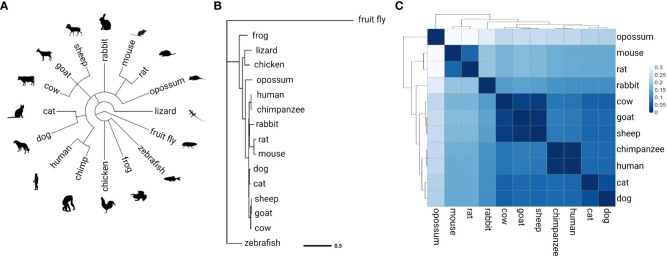
AIB1/NCOA3 protein evolution. **(A)** Overview of the 16 species analyzed. Common taxonomy tree assembled from NCBI Taxonomy Database. **(B)** Phylogenetic tree of AIB1/NCOA3 protein. Branch lengths represent substitutions per site. **(C)** Heatmap of cophenetic distance between AIB1/NCOA3 proteins in all mammals analyzed.

AIB1 protein conservation was examined by assembling a phylogenetic tree containing 16 species (**Figure 5**). Human AIB1 was most similar to orthologous NCOA3 found in the chimpanzee and other mammals and most divergent from zebrafish and fruit fly orthologs. A distance heatmap in **Figure 5C** depicts the cophenetic distances between all mammals analyzed for easy comparison. This value represents the number of substitutions per site and can be interpreted as the amount of genetic change that has occurred during separate evolution of the species compared. The cophenetic distance between human and mouse proteins was 0.158. TATA-Box Binding Protein (TBP) evolutionary conservation was used to compare to a highly conserved protein (67–70) in the same 16 species and had a cophenetic distance of 0.115 between human and mouse, less than a 1.5-fold difference relative to AIB1/NCOA3, indicating the high amount of AIB1/NCOA3 conservation over evolutionary time.

## Function of AIB1 in normal mammary physiology and pathology

Several studies have demonstrated AIB1’s pleiotropic effects in normal physiology. These processes include cell proliferation, survival and metabolism, along with vasoprotection, female reproductive function and puberty (71, 72). Although earlier studies suggested overlapping functions between the three SRC proteins, essential and non-redundant roles for AIB1 were established through the use of knockout mice. AIB1 null mice displayed delayed puberty, slowed mammary gland growth, reproductive malfunction and dwarfism due to alterations in the IGF-1 (insulin-like growth factor 1) signaling pathway (71, 73).

AIB1 was shown to control energy homeostasis through PPARγ coactivator-1α (PGC-1α) signaling (74). AIB1 regulates the expression of the PGC-1α acetyltransferase GCN5 which leads to PGC-1α acetylation and inhibition of its activity. AIB1 null mice displayed increased mitochondrial function and energy expenditure. AIB1 itself is also regulated by a metabolic enzyme. PFKFB4 activates AIB1 through serine 857 phosphorylation (58). Once phosphorylated, AIB1 has increased coactivator interaction with the transcription factor ATF4. This leads to upregulation in the expression of enzyme transketolase (TKT) which directs glucose flux towards the pentose phosphate pathway and purine synthesis. Additionally, AIB1 can form a complex with steroid receptors and PELP1 (proline, glutamic acid, leucine-rich protein 1) in the cytoplasm of a cells (75). This interaction leads to upregulation of HIF-activated metabolic target genes *PFKFB3* and *PFKFB4* which affects mitochondrial respiration and glycolysis.

AIB1 also supports the maintenance of embryonic stem cell pluripotency through regulation of essential pluripotency genes such as Klf4, Tbx3 and Dax-1 (76). Additionally, AIB1 plays a role in immunity which is in part due to its interaction with the inflammatory regulator NF-κB (reviewed in 77). A recent study by Han et al. demonstrated that cell-specific knockout of AIB1 in regulatory T (Treg) cells led to eradication of mouse mammary cancer E0771 cells *in vivo* (78). Mechanistically, AIB1 KO Tregs generated antitumor immunity by enhanced tumor infiltration of effector T cells and natural killer cells while also blocking the immune suppressive function of WT Tregs.

AIB1 has most notably been studied for its role as an oncogene, where it is known to be amplified and/or overexpressed in a variety of cancers including breast and pancreas (23, 37, 79, 80). *NCOA3* is amplified in 5% to 10% of human breast cancers and mRNA is found overexpressed in approximately 30% to 60% of breast cancer cases (23, 79, 81). AIB1 has been shown to promote cancer development through both hormone-dependent and hormone-independent pathways (37, 59, 82, 83). AIB1 overexpression is associated with worse disease-free survival (84) and AIB1 transgenic mice presented with abnormal mammary gland development and mammary adenocarcinomas (82). These mice also displayed high frequency of other tumors such as pituitary and uterus. On the contrary, loss of the oncogene reduced tumor incidence in several BC mouse models (85, 86).

AIB1 advances breast cancer progression though pro-metastatic mechanisms. AIB1 knockout mice containing the mouse mammary tumor virus-polyomavirus middle T (PyMT) transgene had significantly less lung metastasis compared to WT mice (87). Tumors from AIB1 KO mice maintained epithelial markers such as E-cadherin and had lower expression of matrix metalloproteinase 2 (MMP-2) and MMP-9. Mechanistically, AIB1 acted as a coactivator for PEA3 and formed a complex on MMP-2 and MMP-9 promoters to enhance their expression. AIB1 also supported epithelial-mesenchymal transition (EMT) in MCF7 cells, as knockdown of AIB1 upregulated protein and mRNA expression of the epithelial marker E-cadherin and downregulated protein and mRNA expression of the mesenchymal marker Snail (88). The downregulation of E-cadherin (*CDH1* gene) has been shown to be dependent on the interaction of AIB1 with MTA2 and the formation of a repressive complex (89). AIB1 and MTA2 colocalized to the CDH1 promoter in aromatase inhibitor-resistant LetR cells generated from parental MCF7 cells. Knockdown of either AIB1 or MTA2 in these cells caused an increase in *CDH1* expression. This study demonstrated a novel molecular mechanistic link between AIB1 and *CDH1*.

The AIB1Δ4 isoform has also been implicated in breast cancer metastasis. AIB1Δ4 can act as an adapter protein to bridge the epidermal growth factor receptor (EGFR) and focal adhesion kinase (FAK) proteins consequent to EGF stimulation (90). This interaction then potentiates cell migration. In addition, AIB1Δ4 expression has recently been shown to enhance invasiveness of breast cancer cells through cell-cell crosstalk (52, 53). A minor subset of breast cancer cells that express AIB1Δ4 *in vivo* can enable bulk tumor cells to metastasize through alteration in signaling pathways that are activated *via* direct cell-cell contact.

## AIB1 in the regulation of estrogen-dependent effects on breast cancer development and progression

Estrogen drives the proliferation of mammary epithelial and breast cancer cells. Cell cycle advancement is controlled by the CDK (cyclin-dependent kinase) family of serine/threonine kinases and activation of their regulatory cyclins (reviewed in 91). Cyclin D-CDK4/6 activity triggers progression through the G1 restriction point by phosphorylating and inactivating RB (retinoblastoma protein), leading to transcription of cell cycle-progression genes through E2F family transcription factors. The cyclin D1/CDK4/6/RB/E2F1 pathway is often activated in ER positive BC through ERα binding to the cyclin D1 promoter and upregulating its expression. Importantly, AIB1 has been shown to enhance E2-induced expression of cyclin D1 (92, 93). AIB1 is also required for the E2-mediated expression of E2F1 through the recruitment of the methyltransferase CARM1 (94). Beyond its role in regulating cyclin D1 and E2F1 expression, AIB1 has also been shown to be essential for estrogen-dependent growth of MCF-7 cells by enhancing the ability of estrogen to inhibit apoptosis (37). Overexpression of AIB1 or AIB1Δ4, along with ERα, led to abnormal growth responses in epithelial and stromal cells *in vivo* and more rapid formation of early stage BC (95). These responses were greater with the AIB1Δ4 isoform compared to full-length AIB1.

Estrogen also increases expression levels of the PR and the PR modulates ERα action in BC (reviewed in 96). PR response genes can be used as a readout of ER activity. The PR has been shown to increase mammary epithelial cell proliferation through both cyclin D1 -dependent and -independent mechanisms (reviewed in 96). The PR agonist progesterone induces proliferation in PR+ cells in a cell-autonomous manner and also in a paracrine manner through receptor activator of nuclear factor-κB (RANK) and RANK ligand (RANKL) signaling (97). Ligand-bound PR stabilizes RANKL mRNA, leading to elevated protein levels. RANKL then activates NF-κB signaling in neighboring cells through its receptor RANK. The risk of BC increases by 16% when levels of progesterone are raised in postmenopausal women (98). Notably, siRNA knockdown of AIB1 led to a decrease in E2-induced PR expression. Mechanistically, AIB1 binds to the ER at the promoter region of the PR gene to facilitate its transcription (93). Furthermore, AIB1, and especially AIB1Δ4, are strong coactivators of the PR itself (49) with AIB1 being the main coactivator for PR in breast tissue (99). Treatment of human breast cancer cells with the PR agonist medroxyprogesterone acetate led to an enhancement in the interaction between AIB1 and PR and a recruitment to cyclin D1 and Myc promoters (100). Tyrosine phosphorylation of AIB1 at Y1357 has been shown to regulate AIB1’s interaction with several transcription factors, including the PR and ERα (55). Interestingly, treatment of breast cancer cells with E2 induces phosphorylation at this site.

Several studies have probed the association between AIB1 and PR expression levels with somewhat conflicting results (79, 81, 95, 101–104). Bautista et al. analyzed 1157 breast tumors and observed AIB1 gene amplification in 4.8% (79). AIB1 levels were correlated with larger tumor size and ERα and PR positivity. On the other hand, Bouras et al. examined 93 breast carcinomas and discovered a lack of association between AIB1 and ERα or PR expression (81). Still further, overexpression of the AIB1Δ4 splice isoform was shown to enhance PR expression *in vivo* while full-length AIB1 overexpression did not (95). Differences in relative isoform expression levels, which is not determined in most studies, could help explain in part the differences in the earlier findings.

The androgen receptor (AR) is a nuclear receptor that is activated by its ligand testosterone or 5-α-dihydrotestosterone. AIB1 interacts with AR through its first and third LXXLL motif and has been shown to be the favored coactivator for ligand-activated AR (105). Interestingly, several AR mutations have increased binding with AIB1 compared to WT AR. AR has an established function in the progression of prostate cancer and there is evidence that it also plays a role in breast tumor development and progression. A recent study found that there is competition between AR and ERα for interaction with AIB1 in BC cell lines (106). Consequently, there is a reduction in E2-induced cyclin D1 protein, mRNA and gene promoter activity when ligand-activated AR sequesters AIB1. These effects are negated when AIB1 is overexpressed, demonstrating how overexpression or amplification of AIB1 in ER+ BC supports E2-induced tumor cell proliferation in the presence of competing nuclear receptors.

## AIB1’s involvement in endocrine therapy resistance

Aromatase inhibitors, selective estrogen receptor modulators (SERMs) and selective estrogen receptor downregulators (SERDs) are all endocrine therapy approaches used in the adjuvant and metastatic setting to decrease estrogen signaling in ER+ BC (reviewed in 4). Both SERMs and SERDs hinder interactions between the ER and coactivators including AIB1 but through different mechanisms. Similar to other nuclear receptors (13, 107–112), the LBD of ERα contains 11 alpha helices (H1, H3-H12) folded into a three layered antiparallel α-helical sandwich. The central core layer contains three α-helices, H5/6, H9, and H10, sandwiched between two additional layers of helices composed of H1-4, H7, H8, and H11. The central core of the LBD is flanked by H12 (111, 112). SERMs function by blocking coactivator recruitment through binding to the LBD and relocating H12 into the coactivator-binding cleft (reviewed in 113, 114). SERDs, on the other hand, preclude the formation of the coactivator docking site by creating a disordered structure for H12. The different conformations of the LBD induced by the various ligands then leads to differential recruitment of coregulators (reviewed in 115, 116). This differential coregulator recruitment was shown *in vitro* by using multiple synthesized triphenylethylene (TPE) derivatives (117). AIB1 was recruited to the *GREB1* proximal ERE enhancer site at different levels when cells were exposed to the various TPEs.

SERMs are termed selective because they can have estrogenic effects depending on the cellular context. Whether a drug acts as an agonist or an antagonist is determined in part by balance of coactivator or corepressor recruitment to the ER in a particular cell type (118). For example, overexpression of SRC-1 in HeLa cells led to an increase in ER activity in the presence of tamoxifen while overexpression of the corepressor SMRT (silencing mediator of retinoic acid and thyroid hormone receptors) reduced tamoxifen-mediated action. Similar conclusions were drawn from a study looking at coregulator recruitment to ERα in breast (T47D) and endometrial (ECC1) carcinoma cells after exposure to tamoxifen or E2 (119). Coactivators were recruited to genes that were upregulated following treatment while genes that were downregulated were associated with corepressors. Overexpression of either a coactivator or corepressor in these cell lines dictated transcriptional response of ERα-regulated genes, highlighting that the expression level of coregulators determines whether a SERM acts as an agonist or antagonist.

**Figure 6:**
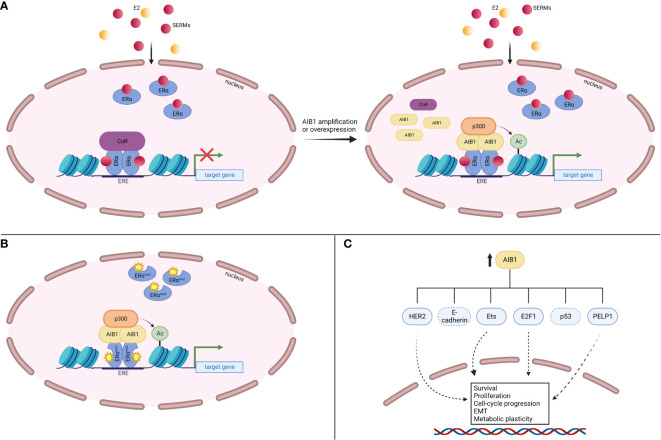
AIB1 contributes to endocrine therapy resistance. **(A)** AIB1 amplification or overexpression can outcompete corepressors (CoR) for binding to SERM-bound ERα, leading to gene expression. **(B)** Mutations in the LBD of ERα can interact with AIB1 in a ligand-independent manner, leading to gene expression. **(C)** AIB1 is involved in several ERα-independent signaling pathways that lead to increased cell survival, proliferation, cell-cycle progression, epithelial-mesenchymal transition (EMT) and metabolic plasticity.

A common mechanism of endocrine therapy resistance involves ligand-independent ER reactivation. This process can happen through several avenues, including altered interactions with coregulators (**Figure 6A**). It has been shown that AIB1 mRNA and protein levels are significantly elevated in normal and malignant tissue after exposure to tamoxifen, even at low dose (120). Conversely, E2 treatment represses AIB1 mRNA and protein expression in MCF-7 cells (121). Knockdown of AIB1 in invasive ductal carcinoma BT474 cells restored tamoxifen antitumor effects, demonstrating AIB1’s role in treatment resistance (122). When analyzing patients with breast cancer who received tamoxifen, those who had high levels of AIB1 protein or mRNA had a worse disease-free survival and higher incidence of tumor recurrence than those who had lower levels of AIB1 (101, 123).

Another path to endocrine therapy resistance is through ERα activating mutations. Mutations often occur in the ER LBD and lead to ligand-independent receptor activity after aromatase inhibitor treatment (reviewed in 124). These mutant ERα proteins are held in an agonist confirmation and their constitutive activity is associated with their ability to interact with coregulators such as AIB1 (125, 126) (**Figure 6B**). Indeed, all three SRC proteins had greater recruitment to EREs when bound by LBD ERα mutants compared to WT apo ERα (127). Missense mutations Y537S and D538G occur in the loop between helix 11-12. The NRD of AIB1 was shown to be able to bind to each of these mutant confirmations without the need for ligand binding (126).

In addition to ERα-dependent mechanisms, AIB1 can lead to endocrine therapy resistance through activation of other signaling pathways (**Figure 6C**). The HER2 (also known as ERBB2) receptor controls cell growth and division and can be overexpressed in BC (reviewed in 128). A study looking at 316 patients with axillary node-positive BC revealed a correlation between high AIB1 expression levels and a worse disease-free survival, especially in patients who had both AIB1 and HER2 overexpression (101). Similarly, Kirkegaard et al. found that AIB1 expression levels are a predictor of relapse following tamoxifen treatment in HER2-expressing BC (102). Knockdown of AIB1 in HER2-amplified cells reestablished tamoxifen’s inhibition on cell proliferation (122). Similarly, silencing AIB1 in a tamoxifen-resistant MCF-7 cell line revealed reduced cell growth through the HER2 signaling pathway and a restoration of tamoxifen sensitivity (129). Mechanistically, AIB1 has been shown to compete with PAX2 (paired box 2 gene product) for binding to the *HER2* cis-regulatory element in the presence of tamoxifen (130). PAX2 binding leads to gene repression whereas AIB1 binding results in an increase in *HER2* transcription. Gene amplification or overexpression of AIB1 therefore outcompetes binding of PAX2 and leads to a reversal in the antiproliferative effects of tamoxifen. Additionally, expression of the AIB1Δ4 isoform in breast cancer cells fails to recruit ANCO1 to the E2 regulatory site found in the HER2 gene (51). ANCO1 recruitment by full-length AIB1 leads to E2-regulated repression of HER2 gene transcription while AIB1Δ4 reverses this repression. These findings delineate the cross-talk between HER2 and ERα signaling that is orchestrated through AIB1.

Ets proteins are downstream effectors of HER2 signaling and mitogen-activated protein kinase (MAPK)-dependent TFs. Primary BC cells and breast cell lines treated with growth factors showed recruitment of SRC proteins, including AIB1, to the Ets-DNA complex (131). Additionally, there was a positive association between SRC and Ets protein expression and disease recurrence. AIB1 was also shown to be required to promote E2-independent cell proliferation through interaction with the cell cycle regulator E2F1 (132). AIB1 overexpression in T47D cells that were made quiescent by tamoxifen or pure anti-estrogen treatment restored their proliferation, even in the presence of continuous anti-estrogens, through E2F1-responsive genes that are associated with proliferation. Interestingly, the interaction between AIB1 and E2F1 happens through the N-terminus of AIB1 (132) which is absent in the AIB1Δ4 isoform.

More recently, the histone acetyltransferase GCN5 (general control non-derepressible 5) was shown to induce tamoxifen resistance by upregulating the expression of AIB1 leading to a reduction in the stability of the tumor suppressor p53 in MCF-7 cells (133). This AIB1-dependent p53 degradation may be happening through AIB1 up-regulation of TRAF4 (tumor necrosis factor receptor associated-factor 4). TRAF4 protein competes with p53 for binding to the deubiquitinase HAUSP (134). A decrease in p53 deubiquitination results in degradation and a reduction in stress-induced cell apoptosis. These results suggest that AIB1 overexpression may be particularly important in tumors with wild-type p53. Further, AIB1 may increase anti-hormone therapy resistance and enhance breast cancer stem cell activity by promoting metabolic plasticity through interaction with PELP1 (75) and contribute to BC metastasis under these therapy resistant conditions by inhibiting *CDH1* (E-cadherin) (89).

## Targeting AIB1 in breast cancer

The transcriptional activity of ERα is regulated by its ligand-dependent conformation which was demonstrated by discovering peptides that interacted with either E2- or tamoxifen- activated ERα (135). The utility of this finding was evident for treatment of tamoxifen resistant breast cancers by being able to target sites outside of the ligand-binding pocket through creating coactivator mimics. Pyrimidine-based coactivator binding inhibitors later demonstrated the feasibility of targeting the coactivator binding pocket of ERα (136). The hope was that interfering with nuclear receptor-coactivator interactions through targeting the receptor-LXXLL interaction would inhibit the transcriptional activity of ERα (30). However, as outlined above, overexpression of coactivators including AIB1 often occurs in human breast cancer and can lead to ERα-independent cell growth. Therefore, small molecule inhibitors (SMIs) that can directly interfere with coactivator activity holds more therapeutic promise. The cardiac glycoside bufalin was identified in 2014 as an inhibitor of both AIB1 and SRC-1 (137). Bufalin was able to reduce tumor growth in mouse xenograft models of BC by degrading AIB1. Nevertheless, due to bufalin’s known cardiotoxicity, new AIB1 SMIs were sought. The SRC-3 inhibitor-2 (SI-2) was identified in 2016 and caused BC cell death with low nanomolar IC50 values (138). Although SI-2 functions as a potent AIB1 inhibitor, its short half-life *in vivo* restricts its use as a practical therapeutic agent. To circumvent this issue, fluorine atoms were recently introduced to the SI-2 core structure (139). These SI-2 analogs have a significantly prolonged plasma half-life and minimal toxicity while remaining effective at inhibiting progression of breast cancer lung metastasis. Beyond inhibiting SRCs to treat cancer, a SRC small molecule stimulator (MCB-613) was identified that led to cancer cell death through over-activation of SRC transcriptional activity, leading to endoplasmic reticulum stress and high levels of reactive oxygen species (140).

## AIB1 ERα-independent activities

The receptor tyrosine kinase EGFR is activated by EGF and regulates cell proliferation and survival of breast cancer, often dimerizing with HER2. Knockdown of AIB1 levels in breast, lung and pancreatic cancer cell lines diminished growth response to EGF (141). Germline KO of AIB1 prevents growth of HER2 dependent cancer in transgenic models (51, 86). There was also reduced tyrosine phosphorylation of EGFR as well as decreased EGF-dependent phosphorylation of HER2. This data suggests that AIB1 can act as an oncogene in part through controlling EGFR and HER2 activity. Further, AIB1Δ4 was shown to act as an adaptor that links EGFR to FAK (90). This interaction promotes EGF-induced phosphorylation of FAK and c-Src which leads to increased cell migration.

AIB1 is also known to regulate cell response to IGF-1 signaling. IGF-1 functions through binding to the IGF-1 transmembrane receptor. Once bound by its ligand, IGF-1 receptor elicits a response that leads to cell proliferation, tissue differentiation and protection from apoptosis through intracellular signaling pathways (reviewed in 142). Knockdown of AIB1 in MCF-7 cells increased IGF-1-dependent anoikis and thus impacted anchorage-independent growth (143). When AIB1 was overexpressed in transgenic mice, there was an increase in mammary IGF-1 mRNA and serum protein levels as well as activation of IGF-1 receptor downstream signaling molecules (82). On the contrary, knockout of AIB1 in mice resulted in partial resistance to IGF-1 without changing the expression of estrogen- or progesterone-responsive genes (85). An alteration in IGF-1-regulated gene set expression in AIB1 KO mice was responsible for the stunted growth and short stature phenotype (73). These data demonstrate that AIB1 is required for IGF-1-depenedent signaling which is independent of its role in ER signaling. In fact, targeting the IGF-1 downstream Akt/mammalian target of rapamycin (mTOR) signaling pathway has been shown to revert the premalignant hyperplastic mammary phenotype seen in an AIB1 transgenic mouse model (144).

AIB1 is known to act as a coactivator for several transcription factors other than the ER. Besides E2F and Ets interactions described above, AIB1 increases the expression of AP-1 (activator protein 1), TEADs and NF-κB (nuclear factor kappa B) dependent target genes, among others (reviewed in 145). Briefly, AIB1 coactivation of AP-1 promoted transcription of matrix metalloproteinases and increased invasiveness of human breast cancer cells (146). AIB1 interacts with the TEAD family of transcription factors through its N-terminal bHLH-PAS domain (147). AIB1, along with YAP (yes-associated protein 1), form a complex with TEAD that can lead to transcriptional activation or repression through AIB1-dependent recruitment of ANCO1 (148). Additionally, AIB1 is a known coactivator of NF-κB and is involved in NF-κB-mediated gene expression (34). These data demonstrate the wide range of functions AIB1 has through its large protein interaction network. Importantly, these ERα-independent protein interactions can promote the progression of triple-negative BC (TNBC) which is characterized by the lack of expression of ER, PR, and HER-2. Indeed, high levels of expression of AIB1 are associated with poor prognosis in TNBC patients (149). Additionally, a subset of TNBC is driven by the AR (150). AIB1 is a coactivator of the AR (105) and therefore targeting AIB1 in AR positive TNBC could be of potential clinical interest, especially given that there are currently no molecular targets for this subtype.

## Conclusions

ERα has been studied extensively for its role in human breast cancer development, maintenance and progression. Inhibiting the production of E2 or targeting ERα itself (collectively termed endocrine therapy) is a common strategy in treating patients with ER+ BC but treatment resistance is prevalent and patients often experience disease progression. Therefore, there is a need to highlight molecules that interact with and influence ERα signaling to broaden our understanding of the biology of ER+ BC and help decipher and anticipate mechanisms of endocrine therapy resistance. AIB1 is a known potent transcriptional coactivator of ERα that functions through direct contact with the nuclear receptor leading to recruitment of additional coregulators such as p300 and activation of gene transcription. AIB1 influences ERα action by modulating chromatin dynamics.

AIB1 is a highly conserved protein which points to its essential functions throughout evolution. However, AIB1 can act as an oncogene in breast and other cancers through both hormone-dependent and hormone-independent mechanisms and is found amplified or overexpressed in a subset of patients. AIB1 supports tumor cell proliferation in part through enhancing ERα-dependent gene transcription, such as cyclin D1 and the PR. It has been implicated in endocrine therapy resistance through multiple mechanisms. Amplification or overexpression of AIB1 can lead to it outcompeting with other coregulators for interaction with ERα even when ERα is bound to SERMs, causing transcriptional activation of ERα-regulated genes. Additionally, AIB1 is known to bind to mutant ERα in a ligand-independent manner which leads to target gene activation. Finally, AIB1 can lead to endocrine therapy resistance through ERα-independent mechanisms by influencing other signaling pathways that are important in cancer.

**Figure 7:**
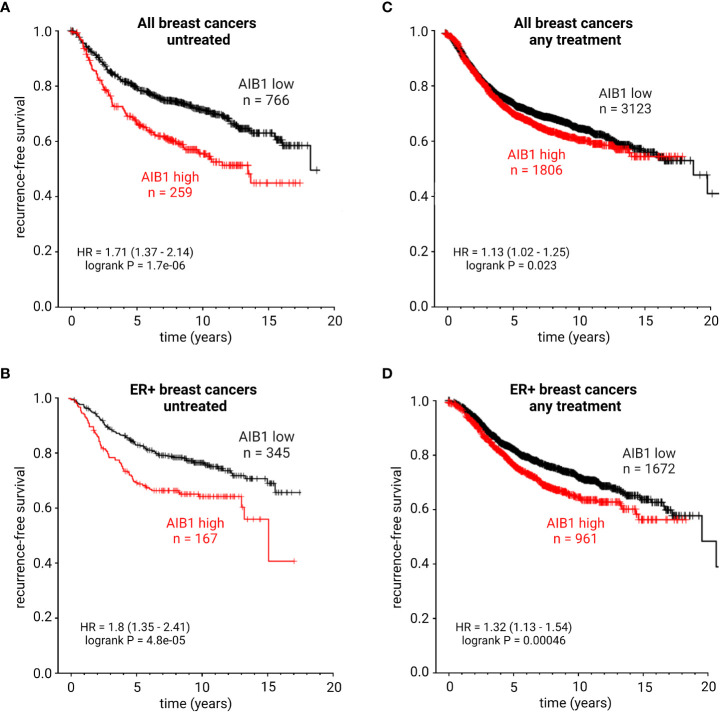
KM plots showing recurrence-free survival probability of patients with all subtypes of breast cancer and untreated **(A)**, ER+ breast cancer and untreated **(B)**, all subtypes of breast cancer with any treatment **(C)**, or ER+ breast cancer with any treatment **(D)**. HR, hazard ratio. Best performing cutoff expression.

AIB1 expression levels can be used as a biomarker in BC (84, 151, 152). High levels of AIB1 are correlated with a more aggressive tumor-phenotype and elevated expression levels after tamoxifen treatment may indicated therapy resistance. To further support these conclusions, AIB1 mRNA expression levels were examined in patients with BC through publically available datasets (kmplot.com). High expression of AIB1 was correlated with a significantly shorter recurrence-free survival (RFS) in both patients with all BC subtypes and also in those with ER+ BC that were untreated (**Figures 7A, B**). Intriguingly, patients with all subtypes of BC or those with ER+ BC receiving treatment but maintaining high levels of AIB1 showed a significantly shorter RFS compared to those patients who had lower levels of AIB1 expression (**Figures 7C, D**). These data point to AIB1 as a potential target to overcome endocrine therapy resistance. A recent study developed a new method of monitoring AIB1 levels in cells through the use of fluorescent-labelled aptamer-functionalized nanomotors (153). Future studies will determine if this method works for detection in tissues.

In conclusion, AIB1 has several ERα –dependent and –independent actions that make it an important molecule in normal human physiology and pathology. Continued effort to define the function of AIB1 in different cellular contexts will provide a framework for understanding its myriad roles in diseases such as breast cancer and help expose tumor cells that are vulnerable to AIB1 inhibition.

## Author contributions

AK wrote the first draft of the manuscript. All authors contributed to manuscript revision, read, and approved the submitted version.
